# A Rare Case of Symptomatic Ciliated Hepatic Foregut Cyst in a 17-Year-Old Female

**DOI:** 10.7759/cureus.43498

**Published:** 2023-08-14

**Authors:** Altay Aliyev, Arturan Ibrahimli, Natavan Azizova, Nijat Alishev, Eldar Ahmadov

**Affiliations:** 1 Oncology, Liv Bona Dea Hospital, Baku, AZE; 2 Oncology, Liv Bona Dea Hospital, Ankara, TUR; 3 Invasive Radiology, Liv Bona Dea Hospital, Baku, AZE; 4 General Surgery, Yeni Klinika, Baku, AZE

**Keywords:** ptc biopsy, ciliated pseudostratified columnar epithelium, benign hepatic lesion, hepatic cyst, hepatic tumor, foregut cyst, hepatic cyst differential

## Abstract

A ciliated hepatic foregut cyst (CHFC) is a very uncommon cystic hepatic lesion that arises from an embryonic remnant of the foregut epithelium. CHFC is predominantly asymptomatic and is found incidentally. However, it can show various clinical presentations such as pain and weight loss. We present the case of a 17-year-old female who came to our hospital with complaints of right subcostal pain and abdominal discomfort and was diagnosed with CHFC by biopsy. Successful laparoscopic resection was performed due to the possibility of malignant transformation. Even though the majority of the patients are asymptomatic and cysts are commonly found incidentally, when the lesion has concerning features, they may need follow-up or resection due to rare reported cases of malignant transformation.

## Introduction

A ciliated hepatic foregut cyst (CHFC) is a rare benign hepatic lesion and is commonly considered a result of the evagination of the embryonic remnants of the aberrant foregut epithelium. The term was first coined by Wheeler and Edmonson in 1984 [1]. Since then, over 100 cases were diagnosed worldwide [2]. CHFC is most commonly found in the left lobe of the liver, specifically in segment IV B. However, in several reports, the CHFC is localized in the right lobe of the liver and the gallbladder wall [2-6]. The cysts are often small (< 4 cm), unilocular, and subcapsular [2-6]. Clinical presentation of CHFC is generally asymptomatic, and the lesion is frequently found incidentally on radio imaging [6-10]. It can also demonstrate various clinical presentations such as pain and weight loss [11]. The management of CHFC is not well established due to its rarity. Here, we present a young female with right upper quadrant pain and abdominal discomfort, who was diagnosed with CHFC by biopsy and underwent laparoscopic excision of the cyst.

## Case presentation

**Figure 1:**
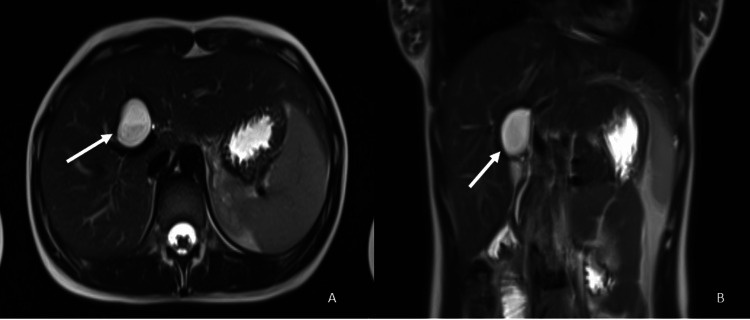
Preoperative contrast-enhanced upper abdomen MRI: (A) T-2 weighted sequence axial view, (B) coronal view. The arrow represents the cystic lesion in IV A segment of the liver.

A 17-year-old female presented to the clinic with a history of severe right upper quadrant pain and abdominal discomfort. The blood workup including liver function tests was found normal. Abdominal computed tomography (CT) scan showed a 2.4 x 2.8 cm precisely contoured hypoattenuating lesion in the right periportal region of the liver. The lesion was circular with a cystic structure filled with dense fluid and was located close to the posterior wall of the right hepatic artery, resulting in compression of the right portal vein. The lesion was further characterized by contrast-enhanced abdominal magnetic resonance imaging (MRI) (Figure 1), which showed the lesion in segment IV A of the liver, in the neighborhood of the gallbladder. 

**Figure 2:**
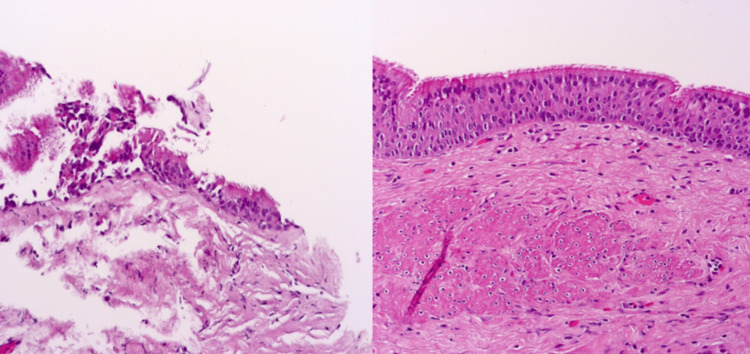
Histology of CHFC with H&E staining (A) Percutaneous transhepatic biopsy of the lesion showing ciliated pseudostratified columnar epithelium and subepithelial connective tissue. (B) Laparoscopic excision confirmed cyst wall lined by ciliated pseudostratified columnar epithelium and smooth muscle bundles in the subepithelial connective tissue, consistent with CHFC (low and high power view respectively). CHFC: ciliated hepatic foregut cyst

The size of the lesion was 3.5x2.4 cm with no enhancement of contrast and no diffusion restriction. The features were consistent with liver cystadenoma. However, based on its location and imaging characteristics on CT and MRI, a definitive diagnosis could not be made, and underlying malignancy could not be ruled out. After the patient was discussed in the tumor board considering her age and the family`s concerns about malignancy, a biopsy decision was taken. Core biopsy was performed by using percutaneous transhepatic cystography (PTC). Histopathological examination revealed the presence of a cyst lined by ciliated pseudostratified epithelial cells, consistent with CHFC (Figure 2). Considering the facts such as the rarity of CHFC, its potential for malignant transformation, the patient's young age, and her active symptoms, laparoscopic cystectomy was decided and performed with the consent of the patient and the family. The surgery and postoperative recovery phase were uneventful. Follow-up examinations were unremarkable. 

## Discussion

CHFC was first described by Friedreich in 1857 [12], but Wheeler and Edmonson defined the major pathological differences between CHFC and cystadenoma, which shares some typical pathological features with CHFC [1]. The unique pathological properties of CHFC classically consist of four layers, including a ciliated pseudostratified columnar epithelium, subepithelial connective tissue, a smooth muscle layer, and an outer fibrous capsule [1,2,13]. These features distinguish the CFHC from cystadenoma and make it similar to ciliated bronchial and esophageal cysts, considered the embryonic remnant of the aberrant embryonic derivative of foregut epithelium [1].

Previously published reports show that there have been approximately 100 cases during the last 150 years, in only a few cases of which patients were symptomatic [6]. The most common presentation of CHFC is asymptomatic, and the lesion is generally found incidentally during routine radiologic imaging, surgery, or autopsy [14]. The mean age of diagnosis is 50 years, with a slight increase in male predominance [6,15].

The radiologic features of CHFC were previously described as small (<4cm), unilocular, and avascular lesions generally found in the medial segment of the left hepatic lobe (segment IV A and IV B), with various elements of the cyst, including clear serous to white or brown material with various densities [1,6-9]. CHFC is commonly identified in ultrasound as a well-defined anechoic or hypoechoic small mass [8]. In addition, CHFC is hypoattenuating, but it can also be iso- to hyperdense on CT [16]. However, on MRI, they are T2 hyperintense and with variable features on T1, with a possible fluid-fluid layer because of the fatty or protein-rich contents of the cyst [7,16]. Based on their radiologic appearance, the differential diagnosis for CHFC could be other unilocular hepatic lesions, such as simple hepatic cyst, echinococcal cyst, epidermoid cyst, intrahepatic choledochal cyst, hepatobiliary cystadenoma or cystadenocarcinoma, hypovascular solid tumor, and others. In our case, the CT scan of the abdomen revealed a 2.4 x 2.8 cm precisely contoured lesion with hypoattenuating features in the right periportal region of the liver parenchyma. Contrast-enhanced abdominal MRI was also performed to make the differential diagnosis, but unfortunately, a definitive diagnosis could not be made with radio imaging. Therefore, a definitive diagnosis was made with a biopsy.

Management of CHFC is controversial because most of the lesions are found incidentally, and patients are generally asymptomatic. Some recommend only observation [17], while others suggest an aggressive approach, like aspiration or surgical resection, due to 5% reported cases of malignant transformation [9-11,13,18,19]. Based on recent articles that report the malignant transformation of CHFCs, serial imaging may be required. Overall, most authors agree that CHFCs have to be surgically resected if the size of the cyst is larger than 4-5 cm, the patient is symptomatic, there is a presence of interval growth, and there are asymptomatic lesions with concerning findings on imaging [6,11,13,18]. Size is the most critical risk factor for the malignant transformation of a CHFC; it has been shown that lesions that are larger than 12 cm are commonly malignant [20]. 

Since it determines the prognosis and management of the patient, a biopsy should be recommended for suspicious lesions that are detected incidentally on systemic screening [7]. Similarly, in young cases, as in our case, it is more appropriate to diagnose by biopsy before deciding on surgery. In our case, laparoscopic removal of CHFC was performed but there are examples of the open surgery approaches in the literature. The type of surgery should be decided based on the facts such as the size of the cyst or the experience of the operating surgeon. We assume, considering the low prevalence, common clinical features, patient's mean age, and sex, that our case is an important contribution to the literature. In addition, the diagnosis with a successful PTC biopsy followed by successful laparoscopic excision is the feature that should be emphasized in our case.

## Conclusions

CHFC is a rare lesion located in the liver. Even though the majority of the patients are asymptomatic and cysts are commonly found incidentally, when the lesion has concerning features, they may need follow-up or resection due to rare reported cases of malignant transformation.
